# Fight or flight alternative mating tactics may explain the iconic male polymorphism of the European stag beetle

**DOI:** 10.1038/s41598-024-73500-x

**Published:** 2024-10-21

**Authors:** Daniele Giannetti, Enrico Schifani, Enrico Rolli, Emanuele Fior, Benedetta Pasquali, Alessandro Campanaro, Donato A. Grasso

**Affiliations:** 1 Department of Chemistry, Life Sciences and Environmental Sustainability, University of Parma, Parma, Italy; 2 Management Authority for Western Emilia’s Parks and Biodiversity, Collecchio, Italy; 3 Research Centre for Plant Protection and Certification, Council for Agricultural Research and Economics, Florence, Italy

**Keywords:** *Lucanus cervus*, Male-male competition, Behavioral ecology, Intrasexual polymorphism, Flight aggregations, Saproxylic beetles, Zoology, Animal behaviour

## Abstract

The existence of different male morphologies within a single species is associated with alternative mating tactics across different animal groups, offering textbook examples of evolutionary biology. The European stag beetle *Lucanus cervus* is a flagship species which has long fascinated naturalists for its charismatic appearance and behavior, with males possessing large mandibles used in ritualized ground fights for females. Males are polymorphic, with larger males possessing disproportionately larger mandibles and being better fighters, while smaller males require less food to develop and may have better chances to escape predation. We discovered an alternative mating tactic that opens a new interpretation on the evolution of the smaller males, based on aggregations of flying males competing to catch flying females in mid-air. Flight aggregations occur earlier in the season and in sites with sparser canopy cover as compared to ground fight aggregations, but most importantly, smaller males tend to prevail due to their greater agility in flight. Competition between males is crucial for reproductive success in this species, and alternative mating tactics involving flight were unknown in beetles, advocating for further research on the ecology and evolution of the iconic male polymorphism of stag beetles.

**Supplementary Information:**

The online version contains supplementary material available at 10.1038/s41598-024-73500-x.

The intriguing evolutionary trade-off represented by secondary sexual characters of animals has long fascinated naturalists and evolutionary biologists, with insects offering several prominent case studies. Stag beetles (Coleoptera, Lucanidae) often exhibit remarkable sexual dimorphism, as males possess exaggerated weaponry in the form of very large mandibles^1^. This can be paired with male polymorphism: adult male size and proportions (which do not vary with age) often follow a gradient of significant allometric variation, with larger males having disproportionally larger mandibles. It is the case of the European stag beetle *Lucanus cervus*^ (Linnaeus, 1758)2^, the largest saproxylic beetle of Europe, characterized by a charismatic appearance and behavior, and, considered a flagship species protected under the European Habitats Directive. Differences between smaller and larger males are thought to play a significant role in the reproductive success of stag beetles^2,3^. Males may greatly outnumber females and face intense competition for reproduction^3,4^. Larger males with larger mandibles win fights for females more often^2,3,5^ (Fig. 1H), while smaller males consume less food as larvae and are more agile flyers^6,7^.

Based on a behavioral field-study focusing of mating aggregations of *L. cervus* conducted in Italy, we documented an alternative mating tactic^8^ that favors smaller males instead of larger ones due to their better flight agility despite lesser fighting success.

We recorded 28 mating aggregations involving a total of 86 males and 28 females. In 17 cases, aggregations comprised males (3 to 6 individuals) competing through fights on the ground in areas with a diameter of approximately 1 to 2.5 m. Fight aggregations lasted 4 to 11 min, until a female approached them by walking closer and was grabbed by one of the males. In the remaining 11 cases, we observed flight aggregations of more numerous males (10 to 30 individuals) performing concentric flights in swarms with a diameter of approximately 1.5 to 3 m and a height of 2 to 16 m (Movie S1). Flight aggregations lasted 3 to 11 min, until they were approached by a flying female which was subsequently grabbed in mid-air by one of the males, the couple immediately falling to the ground, sometimes followed by other males (up to 3) that occasionally tried to interfere. Both fighting and flying aggregations ended with a ‘winning’ male performing a form of mate guarding: winners used their mandibles to hold the thorax of the female, safely transporting her to an area without competing males before mating (Fig. 1I, Movie S2). Other males would sometimes attempt to slip between the two partners during the process: this behavior was observed four times, two of which were entirely unsuccessful, while two times the copula was interrupted prematurely but the interfering male did not mate afterward. Compared to fight aggregations, flight aggregations resulted in smaller males being the winners (Wilcoxon rank-sum test, W = 144.5, *p* = 0.017; Fig. 1A), despite no significant differences in the overall size of participating males (W = 765, *p* = 0.300; Fig. 1B) or females (W = 60.5, *p* = 0.126; Fig. 1C). Both the relative weapon size (mandible length/total length) and the type of mating tactic (fight vs. flight) had a significant influence on the likelihood of mating success for males (logistic regression model, *p* = 0.003 and *p* = 0.014, respectively; Fig. 1D). Furthermore, the interaction between these two predictors was significant, as larger weapon size promoted success in fight aggregations and having a negative impact on success in flight aggregations (logistic regression model, *p* = 0.020; Fig. 1D). In flight aggregations the “mate guarding” transport of the female by the winning male lasted less (W = 157, *p* = 0.002; Fig. 1F) and the copula lasted longer (W = 31, *p* = 0.003) (Fig. 1G). Finally, flight aggregations occurred in areas with a sparser canopy cover (W = 0, *p* < 0.001; Fig. 1I) and were more frequent earlier in the mating season (W = 167, *p* < 0.001; Fig. 1J). Considering possible morphological advantages, analyses on 70 males detected no relationship between bite force and mandible length or total length (*p* = 0.125 and *p* = 0.499 respectively), but only a very weak negative relationship between bite force and head width (Linear regression model, F_1,68_ = 9.99, *p* = 0.002, R^2^ = 0.11).

Alternative mating tactics appear in many animal taxa, with several documented cases related to size differences in males. In beetles, smaller males may emerge earlier, minimizing competition with larger ones^9^. In *Onthophagus* dung beetles, small males dig galleries to reach females without fighting larger males^10^. Small males of the stag beetle *Prosopocoilus inclinatus* exhibit greater persistence in precopulatory behavior, initiate courtship at higher rates, and grasp females more securely, while larger males have greater success in battle, but experience a greater handicap in locomotion, flight costs, and predation risks^6^.

We show that flight aggregations that reward the abilities of males flying and grasping females in mid-air may offer significant opportunities to smaller *L. cervus* males that are less likely to succeed in fights on the ground but are more agile in flight. According to our analyses, their different performance is not related to the grasping force exercised by the mandibles, but larger weaponry is advantageous for fights and disadvantageous in flight aggregations. Without being surrounded by fighting males, winners seem to spend less time in transporting females away and more time in copula. To the best of our knowledge, mating tactics involving males catching the females in the air were never documented in beetles. Mechanisms promoting such aggregations are unknown, however, as in other beetles, chemical cues released by either males or females may be involved^11^. A sparser canopy, characterizing flight aggregations may facilitate orientation during flight. The predominance of fight over flight aggregations later in the season may be due to a higher energetic cost of flying activities considering that adult *L. cervus* have only limited abilities of feeding. Since we observed that males participating in the two different tactics do not averagely differ in size, and only winners do so, it remains to be clarified whether the same male individuals may participate in both tactics during the season. The mechanisms responsible for initiating the aggregations also remain to be explored, with chemical communication channels likely involved.

Male-male intrasexual competition, rather than male-female size ratio as sometimes suggested^12^, seemingly influenced the size of winning males. The discovery of alternative tactics favoring different male morphologies in *L. cervus* opens a new perspective in the evolutionary interpretation of the iconic male polymorphism and advocates for further research on the relative fitness outcomes of the different morphs in this and in other beetle species. Finally, as male polymorphism in *L. cervus* exhibits a notable variation across the species’ vast distribution^13^, different populations may differ in the adoption of alternative mating tactics.

**Fig. 1:**
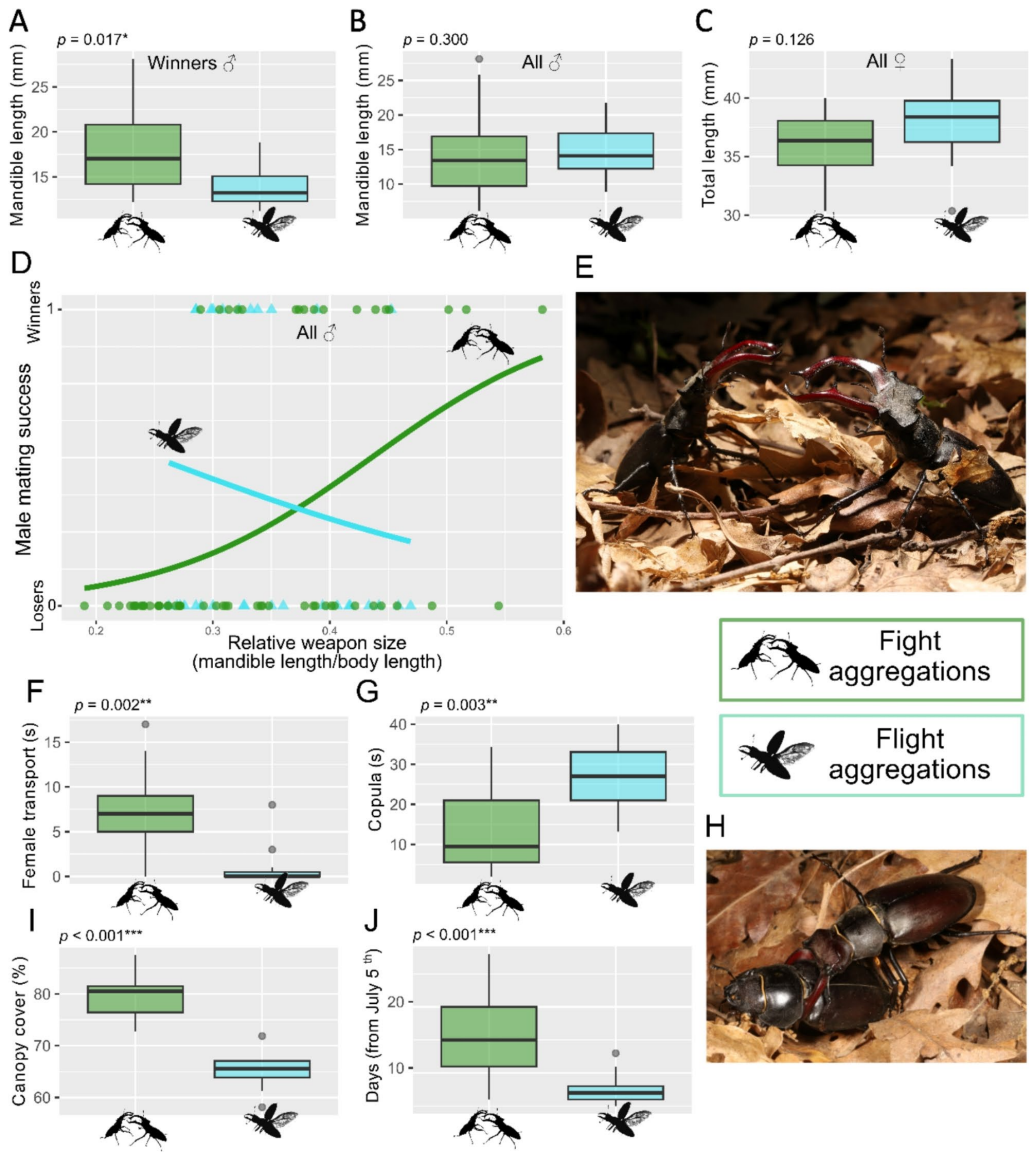
(**A**–**J**) fight and flight aggregations compared: winner males’ size (**A**), size of all males collected (**B**), female size (**C**), combined effect of relative weapon size and aggregation type on mating success (**D**), duration of female transport (**F**), of copula (**G**), canopy cover (**I**), and number days from the beginning of the observations after which aggregations were observed (**J**). Asterisks indicate levels of statistical significance (*** *p* < 0.001, ** *p* < 0.01, * *p* < 0.05). Images of males about to engage in a fight (**E**) and a winner male landed with a female after a flight aggregation (**H**).

## Methods

### Study area

European stag beetles were surveyed near Parma (Emilia-Romagna, Italy), in a site known to host an exceptionally abundant population^4^ (Boschi di Carrega Regional Park). The area has been protected since 1982 and corresponds to a Special Area of Conservation (SAC IT4020001), pursuant to the EU Habitats Directive. Within this Park, we selected the area of ‘Bosco della Capannella’ (44.730389, 10.211556), a 0.15 km^2^ area with an altitude of 130 to 155 m a.s.l. characterized by a deciduous oak forest dominated by *Quercus cerris* L. and *Q. robur* L. This area was subdivided into four different sectors of approximately equal size that were investigated repeatedly one at a time in different days.

### Mating behavior and copula

From 5 July to 5 August 2022, from 7:00 PM until midnight, two researchers investigated one sector per day excluding three days (*n* = 28 days; the remaining three days were dedicated to the activities described in the “Characterization of the bite force of male beetles” section below). Each area was monitored nine times, corresponding to about 39 h of observation per sector. Whenever the researchers found a male and a female *Lucanus cervus* within the range of 3 m, or an aggregation of multiple males within 3 m, they stopped the sector exploration and observed the interactions between stag beetles until either the individuals separated, or until the end of a mating event. The mating behavior was described and quantified recording different parameters: (i) the presence and number of individuals in flight and on the ground; (ii) whether the female was grabbed by the male on the ground or in mid-air; (iii) the time that intercurred from the moment the female was grabbed by a male to the moment they mated, during which the male hold or transported the female (mate-guarding); (iv) number of fights; (v) size the mating males and females and the other males on the ground (this was done only after the copula for what concerns the mating couple, and for the other males on the ground, only once they left the aggregation area, in order to minimize the interference); (vi) the duration of the copula; (vii) the approximate height from the ground and diameter of the aggregation, measured with a laser distance meter. Stag beetles were measured using a caliper. All the standard morphometric characters were measured with the aim of providing a comprehensive characterization of all individuals collected^4,14^: Elytron length (EL), the distance between the scutellum base and the elytra apex (including the scutellum), across the suture; Elytron width (EW), the distance across the pronotal aspect of the elytra; Head length (HL), the distance between the clypeus midpoint and the head base; Head width (HW), the distance between the head ridges above the eyes; Mandible length (ML) with mandibles sealed, the distance between the clypeus and the distal tooth of the apical fork; Pronotum length (PL), the distance across the midline.

### Characterization of the canopy at the mating sites

For each aggregation site we quantified the density of the canopy by using a hemispherical photography technique^15^. A single picture (iPhone 11 with Fisheye Lens Smartphone) was taken perpendicular to the center of the aggregation taking the shot from 1 m using a tripod. The Gap Light Analyser software was used to quantify the canopy coverage.

### Characterization of the bite force of male beetles

To explore the relationship between male size and biting force, we measured the force exerted by the mandibles of *L. cervus* individuals captured outside the aggregations, on five sessions during August-July 2022 from 7:00 pm to 9:00 pm. Bite force was measured minimizing handling time and they were released on the spot. Measurements were taken using an originally designed prototype with Arduino technology (“Insect Pressure Bite Machine – IPBM”), composed of two Arduino board powers with 12 V rechargeable battery packs. Board powers relate to two force sensing resistors (FSR) pressure sensors (left and right) (Response Time: < 1 ms; Restore Time: < 15 ms). Force sensing resistors are conductive polymers exhibiting a decrease in resistance when facing an increase in the force applied. Applying more pressure to the sensor’s head reduces the resistance between its terminals, while releasing the pressure restores the resistance to its original value^5^. The force applied to each FSR was read on the display connected to the Arduino. To convert the values shown in the display into grams, a calibration curve was constructed using standard weights. Known weights were applied to each sensor through a stable bench stand, in 10 gr increments on a scale from 0 to 1,000 gr. An average of 10 readings were applied for each weight. The curve was divided into two parts. For values measured by applying weights from 0 to 50 g (readings in the 0 to 20 range), we obtain a linear function (described by the formula Y = 0.4245X – 2.295) that perfectly interpolates the data obtained (R2 = 0.9968). For readings from 21 to 1,000 g, we obtain a curve described by a second-order polynomial function (Y = 0.0001 × ^2^ + 0.0044X + 27.409), with R2 = 0.991. Using the two equations, it was possible, for each bite taken, to calculate the relative force expressed in grams. During the field bite test, the sensors were covered with a layer of rough paper to offer better adherence and specularly placed on a wooden support (ø 1 cm, height 10 cm). We tested the bite of 70 males and recorded the highest value. As above each male was measured according to Romiti et al.^14^: Elytron length (EL), Elytron width (EW), Head length (HL), Head width (HW), Mandible length (ML), Pronotum length (PL). Body length (BL) was defined as EL + PL + HL.

### Statistical analysis

All statistical analyses were carried out using the software R (4.3.2)^16^. Considering data distribution, Wilcoxon rank-sum tests were used to analyze differences between fight and flight aggregations in mandible length among winning males (mm), mandible length among all males (mm), female total length (mm), female transport duration (s), copula duration (s), percentage canopy cover, and number of days passed since the beginning of the observations (July 5th ). A logistic regression was used to examine the effect of relative weapon size (Mandible length/Body length) and aggregation type (fight or flight) on the likelihood of mating for participating males. Linear regression models were used to explore separately the relationship between mandible length, total male length, and head width with bite force of measured males.

## Electronic supplementary material

Below is the link to the electronic supplementary material.

Supplementary Material 1

Supplementary Material 2

Supplementary Material 3

## Data Availability

All data generated or analysed during this study are included in Supplementary Information files.

## References

[1] Goyens, J., Van Wassenbergh, S., Dirckx, J. & Aerts, P. Cost of flight and the evolution of stag beetle weaponry. *J. R Soc. Interface*. **12**, 20150222 (2015). 25878126 10.1098/rsif.2015.0222 PMC4424704

[2] Lagarde, F., Corbin, J., Goujon, C., Poisbleau, M. & M Polymorphisme Et performances Au combat chez les mâles de lucane cerf-volant (*Lucanus cervus*). Revue d’Écologie. *(La Terre et la. Vie)*. **60**, 127–137 (2005).

[3] Goyens, J. & Dirckx, J. Aerts Stag beetle battle behavior and its associated anatomical adaptations. *J. Insect Behav.* **28**, 227–244 (2015).

[4] Giannetti, D. et al. Grasso A multidimensional study on population size, deadwood relationship and allometric variation of *Lucanus cervus* through citizen science. *Insect Conserv. Divers.* **16**, 638–648 (2023).

[5] Goyens, J., Dirckx, J. & Aerts, P. Jaw morphology and fighting forces in stag beetles. *J. Exp. Biol.* **219**, 2955–2961 (2016). 27436136 10.1242/jeb.141614

[6] Okada, Y. & Hasegawa, E. Size-dependent precopulatory behavior as mate-securing tactic in the Japanese stag beetle, *Prosopocoilus inclinatus* (Coleoptera; Lucanidae). *J. Ethol.* **23**, 99–102 (2005).

[7] Thomaes, A. & Camps, P. Is the major-minor male dimorphism of the stag beetle (*Lucanus cervus*) explained by a weaponry and wing investment trade off? *Bull. Ann. Soc. R Belge Entomolog*. **152**, 152–156 (2016).

[8] Oliveira, R. F., Taborsky, M. & Brockmann, H. J. (eds) *Alternative Reproductive Tactics: An Integrative Approach* (Cambridge University Press, 2008).

[9] Eberhard, W. G. Beetle horn dimorphism: making the best of a bad lot. *Am. Nat.* **119**, 420–426 (1982).

[10] Emlen, D. J. Alternative reproductive tactics and maledimorphism in the horned beetle *Onthophagus acuminatus* (Coleoptera: Scarabaeidae). *Behav. Ecol. Sociobiol.* **41**, 335–341 (1997).

[11] Harvey, D. J. et al. Novel pheromone–mediated reproductive behaviour in the stag beetle, *Lucanus cervus*. *Sci. Rep.* **14**, 6037 (2024). 38472207 10.1038/s41598-024-55985-8 PMC10933271

[12] Harvey, D. J. & Gange, A. C. Size variation and mating success in the stag beetle, *Lucanus cervus*. *Physiol. Entomol.* **31**, 218–226 (2006).

[13] Harvey, D. J., Gange, A. C., Hawes, C. J., & Rink, M. (2011). Bionomics and distribution of the stag beetle, Lucanus cervus (L.) across Europe. *Insect. Conserv. Divers.*, **4**(1), 23–38. 10.1111/j.1752-4598.2010.00107.x

[14] Romiti, F. et al. Latitudinal cline in weapon allometry and phenology of the European stag beetle. *Nat. Conserv.* **19**, 57–80 (2017).

[15] Chianucci, F. & Cutini, A. Digital hemispherical photography for estimating forest canopy properties: Current controversies and opportunities. *IFor. - Biogeosci. For.*. **5**, 290 (2012).

[16] R Core Team. R: A language and environment for statistical computing. R Foundation for Statistical Computing, Vienna, Austria. URL (2024). https://www.R-project.org/

